# 325. Molecular Characterization of Antimicrobial Resistance Genes in *Neisseria gonorrhoeae* in Southeast Michigan

**DOI:** 10.1093/ofid/ofac492.403

**Published:** 2022-12-15

**Authors:** Anita Shallal, Geehan Suleyman, Katherine Gurdziel, Mary Perri, Marcus Zervos, Robert Tibbetts

**Affiliations:** Henry Ford Health, Detroit, Michigan; Henry Ford Health, Detroit, Michigan; Wayne State University, Detroit, Michigan; Henry Ford Hospital, Detroit, MI; Henry Ford Hospital, Detroit, MI; Henry Ford Health, Detroit, Michigan

## Abstract

**Background:**

Antimicrobial resistance (AMR) in *Neisseria gonorrhoeae* (NG) is a public health crisis. Diagnosis of infection is primarily established using nucleic acid amplification technologies (NAATs) without susceptibility testing; thus rapid identification of resistance determinants is crucial. Whole-genome sequencing (WGS) is a promising alternative to current susceptibility methods. The purpose of this study was to describe AMR genes in a subset of isolates using WGS from Southeast Michigan.

**Methods:**

Isolates, demographic data, and minimum inhibitory concentrations (MIC) via E-test (ceftriaxone/CRO) and broth microdilution (azithromycin/AZM) were obtained from the Michigan Department of Health and Human Services. Libraries from isolates were generated using QIAseq FX DNA library kit before sequencing on NovaSeq 6000. Reads were trimmed [Trimmomatic] and aligned to the NG genome [Burroughs-Wheeler Aligner] before processing with Samtools suite for variant detection and consensus WGS determination. AMRFinderPlus was used to categorize AMR genes and resistance-associated point mutations.

**Results:**

Total of 38 isolates were analyzed. The majority were from males (63%) and Blacks (44.7%) living in Detroit City proper (47.3%) with median age 25 (range 13-56), unknown HIV status (55.2%) and sexual orientation (47.3%) (Table 1). Urine (47.3%) was the most common specimen source, followed by cervix/vagina (28.9%). More than a third had prior NG and other sexually transmitted infections. Eight resistance genes were found among the 38 isolates (Figure 1). The penA gene associated with cephalosporin resistance was found in all isolates; however, all isolates were susceptible to CRO (CLSI susceptible breakpoint MIC < 0.25). The mtrR gene associated with macrolide resistance was noted in 86.8% isolates. Among 33 isolates where AZM susceptibility was available, resistance was noted in 9 (27%) (CLSI resistance breakpoint MIC > 1).

**Figure:**
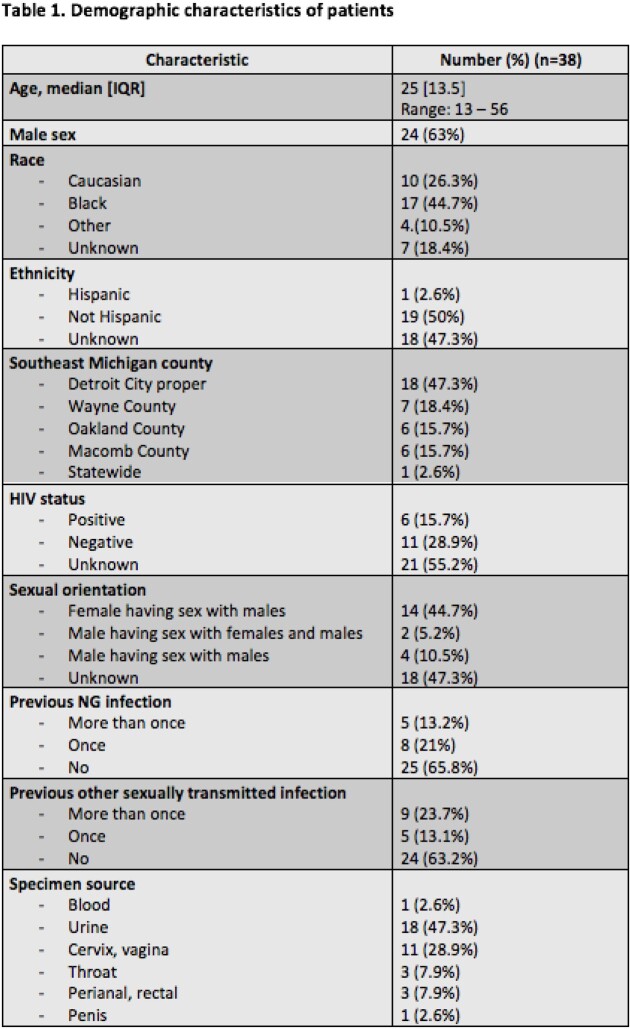

**Figure:**
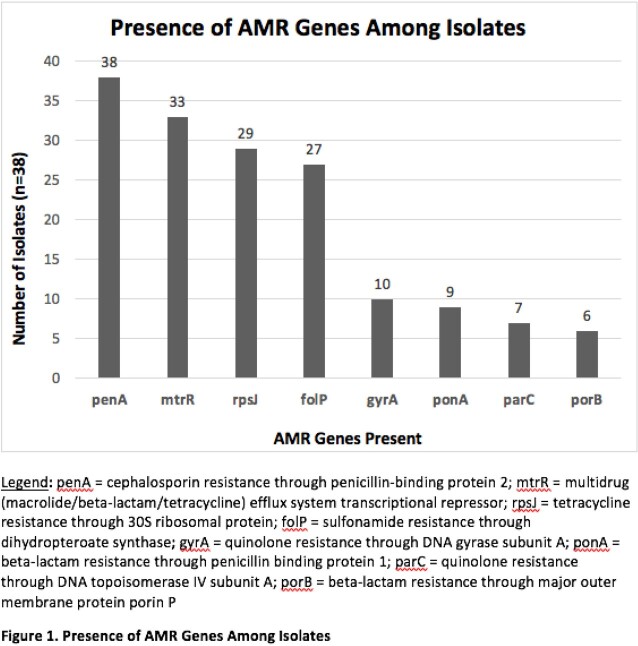

**Conclusion:**

We describe a potential utility for WGS to identify AMR genes in NG. Although numerous β-lactam resistance genes were detected in all isolates, all remained susceptible to CRO. Further research is needed to determine the extent to which genetic diversity affects phenotypic susceptibilities in NG.

**Disclosures:**

**All Authors**: No reported disclosures.

